# Regulation of PGC-1α Isoform Expression in Skeletal Muscles

**Published:** 2015

**Authors:** D. V. Popov, E. A. Lysenko, I. V. Kuzmin, Vinogradova Vinogradova, A. I. Grigoriev

**Affiliations:** Institute of Biomedical problems, Russian Academy of Sciences, Khoroshevskoye shosse, 76A, Moscow, 123007, Russia; Faculty of Fundamental Medicine, M.V. Lomonosov Moscow State University, Lomonosovskiy prospect, 26B–10, Moscow, 119192, Russia; Department of Genetics, Faculty of Biology, M.V. Lomonosov Moscow State University, Leninskie Gory, 1–12, Moscow, 119991, Russia

**Keywords:** alternative splicing, alternative promoter, skeletal muscle, PGC-1α, gene expression

## Abstract

The coactivator PGC-1α is the key regulator of mitochondrial biogenesis in skeletal muscle. Skeletal muscle expresses several PGC-1α isoforms. This review covers the functional role of PGC-1α isoforms and the regulation of their exercise-associated expression in skeletal muscle. The patterns of PGC-1α mRNA expression may markedly differ at rest and after muscle activity. Different signaling pathways are activated by different physiological stimuli, which regulate the expression of the *PGC-1α *gene from the canonical and alternative promoters: expression from a canonical (proximal) promoter is regulated by activation of the AMPK; expression from an alternative promoter, via a β2-adrenergic receptor. All transcripts from both promoters are subject to alternative splicing. As a result, truncated isoforms that possess different properties are translated: truncated isoforms are more stable and predominantly activate angiogenesis, whereas full-length isoforms manly regulate mitochondrial biogenesis. The existence of several isoforms partially explains the broad-spectrum function of this protein and allows the organism to adapt to different physiological stimuli. Regulation of the *PGC-1α *gene expression by different signaling pathways provides ample opportunity for pharmacological influence on the expression of this gene. Those opportunities might be important for the treatment and prevention of various diseases, such as metabolic syndrome and diabetes mellitus. Elucidation of the regulatory mechanisms of the* PGC-1α *gene expression and their functional role may provide an opportunity to control the expression of different isoforms through exercise and/or pharmacological intervention.

## INTRODUCTION

Skeletal muscle constitutes more than 30% of body mass in adults. As skeletal muscles have high levels of metabolic and secretory activity, they are identified as secretory organs that have an influence on other organs [1]. Blood flow and the consumption of oxygen and substrates (glucose, fatty acids, etc.) increase significantly as active skeletal muscles contract. The pronounced accumulation of calcium ions and other metabolites occurs simultaneously in muscle fibers; a decrease in energy charge and redox potential may also occur. Aerobic training induces the following marked adaptive changes in skeletal muscles: capillarization, changes in mitochondrial density, and an increase in the activity of oxidative enzymes. Maximum oxygen consumption and aerobic performance of muscles improve due to these changes. These adaptive changes are tightly connected to the functioning of coactivators belonging to the PGC-1 family (peroxisome proliferator- activated receptor (PPAR) gamma coactivator 1). This family includes PGC-1α, PGC-1β, and PGC-related coactivators. One of these proteins, PGC-1α, plays the most important role in regulation of mitochondrial biogenesis in skeletal muscle.

Several isoforms of PGC-1α exist [2,3]; this partially explains the broad-spectrum function of this protein. Over the past decade, many studies have focused on PGC-1α function, the molecular mechanisms of its activation, and the regulation of *PGC-1α *gene expression. Skeletal muscle expresses several PGC-1α isoforms. This review is devoted to the functional role of PGC-1α isoforms and to the regulation of their expression in skeletal muscle at rest and during recovery after exercise.

## FULL-LENGTH PGC-1α ISOFORMS

**Functional role of PGC-1α**

**Fig. 1:**
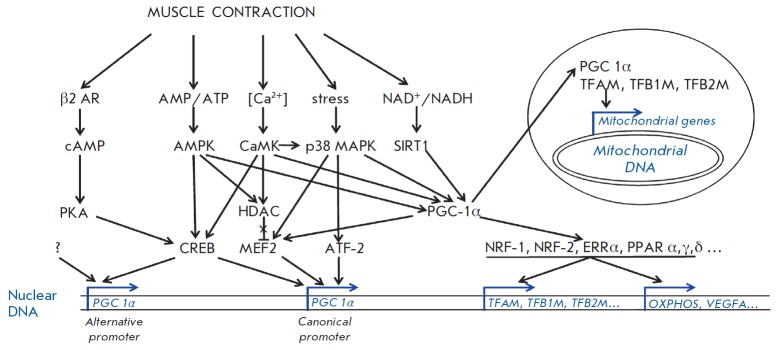
The scheme of PGC-1α protein activation and regulation of the *PGC-1α *gene expression from canonical (proximal) and alternative promoters.*AMPK *– AMP-activated protein kinase, *ATF *– activating transcription factor, *CaMK *– Ca^2+^/calmodulin-dependent protein kinase, *CREB *– cAMP response element-binding protein, *ERR *– estrogen-related receptor, *HDAC *– class IIa histone deacetylase, *MEF *– myocyte enhancer factor, *NRF *– nuclear respiratory factor,* OXPHOS *– oxidative phosphorylation related genes, *p38 MAPK *– p38 mitogen-activated protein kinases, *PGC *– peroxisome proliferator-activated receptor gamma, coactivator, *PKA *– protein kinase A, *PPAR *– peroxisome proliferator- activated receptor, *SIRT1 – *NAD-dependent deacetylase sirtuin-1, *TFAM *– mitochondrial transcription factor A, *TFB1M *–mitochondrial transcription factor B1, *TFB2M *–mitochondrial transcription factor B2, *VEGFA *–vascular endothelial growth factor A, *β2AR *–β2-adrenergic receptor

Several signaling kinases, such as AMPK, CaMK, and p38 MAPK [4] and the NAD-dependent deacetylase sirtuin-1 (Sirt-1) [5], are activated in skeletal muscle during and immediately after acute endurance exercise. This activation results in an increase in *PGC-1α* (*PPARGC1A*) gene expression (see below); an increase in the phosphorylation and acetylation of existing PGC-1α also occurs (i.e., PGC-1α activation) (*Fig. 1*). In rodents [6,7] and humans [8,9], activated PGC-1α translocates from skeletal muscle to the nucleus and coactivates many transcription factors and nuclear receptors. Exercise-induced activation of PGC-1α may occur without increasing the level of this protein in the nucleus. In human skeletal muscle, acute endurance exercise leads to increased AMPKα2 [10] and phosphorylated p38 MAPK [11] levels in the nucleus. The authors assumed that the nuclear translocation of these kinases promotes activation of PGC-1α in the nucleus.

Activated PGC-1α regulates the expression of its own gene via the feedforward mechanism [12]. The activated protein also co-activates the nuclear respiratory factors (NRF) -1 and -2, estrogen-related receptor (ERR) α, and peroxisome proliferator-activated receptors (PPAR) α and γ. The activation of these nuclear receptors and transcription factors induces the expression of many genes involved in the regulation of oxidative phosphorylation (OXPHOS) and fat and carbohydrate metabolism [13-15]. NRF-1 and NRF-2 induce the expression of the mitochondrial transcription factors A (*TFAM*), B1 (*TFB1M), *and B2 (*TFB2M*) genes. These transcription factors, primarily TFAM and TFB2M, translocate to the mitochondria and initiate the expression of genes from mitochondrial DNA [16]. The PGC- 1α–TFAM complex has been found in mitochondria [7,17,18], where it most likely initiates mitochondrial DNA transcription (*Fig. 1*). PGC-1α also induces the expression of the hypoxia-inducible factor (HIF), independent of the vascular endothelial growth factor A (*VEGFA*) gene [19,20]. It has recently been demonstrated that skeletal muscle angiogenesis is connected to the PGC-1α-dependent activation of macrophages [21].

Acute endurance exercise induces a pronounced increase in the expression of the *PGC-1α *gene, the activation of PGC-1α present in the cell, and changes in the intracellular localization of the protein. PGC-1α has a complex effect on nuclear and mitochondrial DNA gene expression. This protein is one of the most important regulators of mitochondrial biogenesis, fat and carbohydrate metabolism, and angiogenesis in skeletal muscle (*Fig. 1*).

**Regulation of PGC-1α gene expression derived from the canonical promoter**

Over a decade ago, Puigserver and coworkers cloned the *PGC-1α *gene from mice [22]. The human *PGC-1α* gene contains 13 exons; this gene encodes a protein composed of 798 a.a. with a calculated mass of ~91 kDa. The promoter of this gene contains two major transcription initiation sites 90 and 119 bp upstream of the initiation transcription codon ATG [23]. The *PGC-1α* canonical (proximal) promoter contains two conservative binding sites for myocyte enhancer factor 2 (MEF2) and one CRE-binding site for the cAMP response element-binding protein (CREB) [23]. The regulation of *PGC-1α *gene expression derived from the canonical promoter was investigated in detail (see below and *Fig. 1*). Cellular models and mice were investigated [12,24]; *in vivo* study of mice skeletal muscle by means of optical bioluminescence was performed [25] and the results confirmed the essential role of MEF2 and CREB in activation of *PGC-1α *gene expression derived from the canonical promoter*.*

Activated PGC-1α can coactivate MEF2, thereby upregulating its own gene expression [11,12]. Acute cycling exercise increases the phosphorylation level of nuclear p38 MAPK^Thr180/Tyr182^, increases the amount of the p38 MAPK^Thr180/Tyr182^ –MEF2 complex in human skeletal muscle [11], and apparently activates MEF2. MEF2 activity is inhibited by class IIa histone deacetylases (HDAC) [26], primarily HDAC5 [25]. Acute endurance exercise increases the phosphorylation level of HDAC5; in turn, this phosphorylation leads to the dissociation of the MEF2–HDAC5 complex and the nuclear export of HDAC5 [11,27]. Endurance exercise-induced phosphorylation of HDACs is regulated by the kinases CAMKII and AMPK [27]; these kinases respond to intracellular levels of AMP and calcium ions [28,29]. The phosphorylation level and/or the activation of CAMKII and AMPK are positively correlated with the intensity of the endurance exercise [4,30-34].

The CRE transcription factor family includes CREB and the activating transcription factor (ATF)-2. Phosphorylation of CREB^Ser133^ and its subsequent activation is regulated by several signaling kinases, including CAMKII and AMPK [35,36]. The activation of CAMKII and AMPK induced by acute endurance exercise increases the phosphorylation level of CREB^Ser133^; the phosphorylation of this protein upregulates *PGC-1α* gene expression [4,37]. The phosphorylation level of CREB^Ser133^ during latter recovery depends on the intensity of the endurance exercise [4].

Stress-mediated activation of p38 MAPK upregulates* PGC-1α *gene expression via phosphorylation of ATF-2^Thr71^ and its subsequent activation [37-39]. Several factors may activate p38 MAPK, including calcium ions and reactive oxygen species [37,39]. Endurance exercise leads to an intensity-independent increase in the p38 MAPK^Thr180/Tyr182^ phosphorylation level [4]. Phosphorylation of p38 MAPK^Thr180/Tyr182^ may be determined by a systemic factor; the p38 MAPK^Thr180/Tyr182^ phosphorylation level increases after acute endurance exercise even in inactive muscle [40]. The phosphorylation level of ATF-2^Thr71^ depends on the intensity of the endurance exercise. This finding indirectly indicates that another signaling pathway may be involved in exercise-mediated phosphorylation of ATF-2^Thr71^ [4].

The exercise-induced activation of various signaling kinases and their targets, including HDACs, MEF2, ATF-2, and CREB, upregulates the transcriptional activity of the *PGC-1α *promoter. Moreover, total PGC-1α mRNA expression is associated with exercise of moderate to maximal aerobic power [4,41-43].

**Regulation of expression of the PGC-1α gene derived from an alternative promoter**

**Fig. 2:**
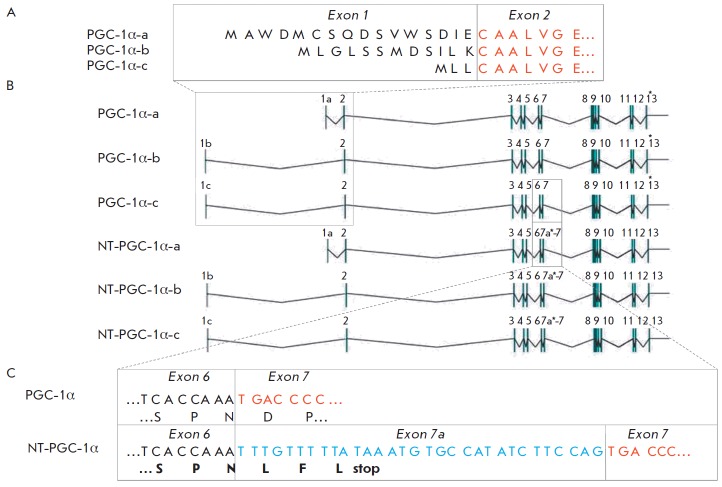
A – Different PGC-1α mRNA isoforms are expressed from canonical (PGC-1α-a) and alternative (PGC-1α-b and PGC-1α-c) promoters in mice and encode different amino acid sequences in the first exon. B – Scheme of exons (vertical line) of different isoforms and their genomic DNA locations. The asterisk is a stop-codon. C – Nucleotide and amino acid sequences between exons 6 and 7 in the full-length (PGC-1α) and truncated (NT-PGC-1α) isoforms

Two groups of researchers have recently independently described an alternative promoter of *PGC-1α *in skeletal muscle, which is located ~14 kb upstream of the canonical (proximal) promoter (*Fig. 2A*) [2,44]. An additional promoter located 587 kb upstream of the canonical promoter has been described in human nerve tissue. This promoter gives rise to several isoforms of PGC-1α mRNA. However, these transcripts were not detected in skeletal muscle [45]. A tissue-specific isoform of* PGC-1α *was also found in liver (L-PGC-1α) [46]. In this review, the focus will be mainly directed at the mechanisms behind the regulation of *PGC-1α *gene expression in skeletal muscle.

Miura *et al*. investigated the effect of β-adrenergic receptor activation on *PGC-1α *gene expression*. *The β2-agonist clenbuterol substantially increased *PGC-1α* gene expression in mice skeletal muscle. The increase was not observed in knockout animals without β1-, β2-, and β3-adrenergic receptors. The β2-blockers propranolol and ICI 118551 tempered the exercise-induced (45 min running, 15 m/min) increase in *PGC-1α *gene expression in the skeletal muscle of wild-type mice [47]. These findings suggest that *PGC-1α *gene expression is regulated at least in part via β-adrenergic receptor activation. The authors also found that different PGC- 1α mRNA isoforms are expressed in skeletal muscle [2].

It has been demonstrated [2,20,44] that new transcripts originate from an alternative promoter located 14 kb upstream of the canonical promoter. The canonical promoter originates at the first exon (1a) of the canonical PGC-1α-a mRNA isoform. Due to alternative splicing, the alternative promoter directs the transcription of two different first exons (1b and 1c), which results in the PGC-1α-b and PGC-1α-c mRNA isoforms, respectively. The nucleotide sequence from the second exon to the 13th exon is identical in the isoforms PGC-1α-a, PGC- 1α-b, and PGC-1α-c. The amino acid sequences encoded by the first exons of PGC-1α-b and PGC-1α-c mRNAs differ and are shorter than that of PGC-1α-a mRNA by 4 and 13 a.a., respectively (*Fig. 2A*). At rest, the mRNA abundance of transcripts derived from the alternative promoter in the skeletal muscle of mice [2,20,48] and humans [49-51] are much lower than that of transcripts derived from the canonical promoter. However, one study demonstrated that the levels of PGC-1α-a, PGC-1α-b, and PGC-1α-c mRNAs were similar in resting skeletal muscles in mice [52].

The proteins encoded by the new transcripts are found to be functionally active. The functional activity of these isoforms was evaluated by transfecting HEK 293 cells with plasmids encoding different nuclear receptor PPAR (α, -d, and -g) and PGC-1α isoforms. The proteins PGC-1α-b, PGC-1α-c, and PGC-1α-a activated PPARs [2]. The physiological significance of expression from an alternative promoter was confirmed using transgenic mice. The overexpression of PGC-1α-b and PGC-1α-c in skeletal muscle led to the activation of OXPHOS-related genes and the genes regulating fat metabolism [2]. Another study revealed that the overexpression of PGC-1α-b in the skeletal muscle of mice induces the expression of PGC-1α target genes, such as cytochrome c oxidase (*COX*) 2 and 4, genes that regulate fat metabolism (i.e., *CD36*, *MCAD*, and *CPT1*), and the angiogenesis-associated gene *VEGFA*; the activity of citrate synthase (CS), a marker of mitochondrial density, also increases. During an incremental treadmill test, transgenic mice exhibited increased aerobic performance, a higher maximal oxygen consumption rate (•Vo_2max_), an increased percentage of oxidized fat, and a lower accumulation of lactate in blood compared with wild-type animals [53].

The expression of PGC-1α mRNA from different promoters is regulated by different stimuli. The voluntary wheel [20] and moderately intensive (15 m/min, 45 min) treadmill [2] running induced a pronounced increase in PGC-1α-b and PGC-1α-c mRNA expression in mice skeletal muscle; however, expression of PGC- 1α-a mRNA derived from the canonical promoter remained unchanged. An increase in the running speed up to 20 and 30 m/min led to a proportional 20- and 33-fold increase, respectively, in the PGC-1α-b mRNA level [48]. Increasing the running speed resulted in only a small rise in the mRNA level of PGC-1α-a derived from the canonical promoter (1.4- and 1.8-fold increase at running speeds of 20 and 30 m/min, respectively). Following these running sessions, the PGC-1α-b mRNA level was higher than the PGC-1α-a mRNA level. A similar ratio was observed between the PGC- 1α-b mRNA level and the PGC-1α-a mRNA level in human skeletal muscle during recovery after moderately intense exercise (45–90 min) [49,50]. The differences in the regulation of the expression of PGC-1α isoforms in response to different physiological stimuli were found in other tissues with high metabolic activity. After 21 h of starvation, only the expression of PGC-1α-a mRNA markedly increased in mouse liver. After exposure to cold temperatures (4°C) for several hours, only expression of PGC-1α-b and PGC-1α-c mRNA increased in the brown adipose tissue of mice [2,54].

The aforementioned studies suggest that the expression derived from the alternative promoter is regulated via activation of a β-adrenergic receptor. This hypothesis was confirmed in the following studies. A clenbuterol injection into the skeletal muscle of mice at rest increased the mRNA levels of PGC-1α-b and PGC-1α-c (PGC-1α-2 and PGC-1α-3, respectively, in Chinsomboon *et al.*) by several orders of magnitude; however, the mRNA level of PGC-1α-a (PGC-1α-1 in Chinsomboon *et al.*) remained unchanged [2,20]. The β-adrenergic receptor inhibitors propranolol and ICI 118551 suppressed the increase in expression from the alternative promoter that is induced by endurance exercise. The pharmacological activation of AMPK was expected to increase the specific expression of *PGC-1α* from the canonical promoter. The agent 5-aminoimidazole- 4-carboxamide-1β-D-ribofuranoside (AICAR) was used to activate AMPK. However, the injection of AICAR into the skeletal muscle of mice upregulated the expression from both the canonical promoter (by ~50%) and the alternative promoter (by tenfold) [48]. The authors assumed that the increase in the expression derived from the alternative promoter might be connected to the AICAR-mediated increase in the blood level of catecholamines and stimulation of β-adrenergic receptors in the muscle. In mice, AICAR treatment increased the plasma concentrations of adrenaline and noradrenaline tenfold and by 30%, respectively. The systemic influence of AICAR was excluded in the experiment with isolated rat epitrochlearis muscle. AICAR treatment increased the expression of PGC-1α-a mRNA by ~50%; however, the expression level of PGC- 1α-b mRNA remained unchanged [48]. This result is in accordance with the findings reported in the previous myoblast study. AICAR treatment did not induce expression from the alternative promoter in C2C12 cells; forskolin, an activator of adenylate cyclase, upregulated expression only from the alternative promoter [44]. Other regulators of expression from the alternative promoter were also revealed. It has been demonstrated that MKK6, a kinase of p38 MAPK, and treatment with calcium ionophore activate expression from the alternative promoter. The constitutively activated forms of the major participants of calcium signaling, CaMKIV and phosphatase calcineurin A, can also upregulate expression from the alternative promoter. The transfection of myoblasts with plasmids containing wild-type or mutant fragments of the alternative promoter confirmed that activation of the alternative promoter depends on the binding of CREB to the CRE site. A similar result was achieved when *m. tibialis anterior *was used to transfect mice [20]. As mentioned above, both the alternative promoter and the canonical promoter contain CRE sites. It remains unclear why phosphorylation of CREB by β-adrenergic receptor signaling induces expression primarily from the alternative promoter [48]. The canonical promoter contains the typical CRE site sequence TGACGTCA (CREB/ATF consensus); the alternative promoter contains a palindromic CRE site with a single nucleotide substitution. This variant of the CRE site can bind CREB and is essential for the initiation of transcription from the alternative promoter. However, the affinity of CREB to a CRE site with a single nucleotide substitution is lower than that of the typical CRE site in the canonical promoter [44,48].

It can be assumed that at rest, even a low concentration of phosphorylated CREB is sufficient to induce high (near maximal) expression from the canonical promoter; the gene expression is induced only slightly as a result of the increased level of phosphorylated CREB. However, a high level of phosphorylated CREB is required to activate transcription from the alternative promoter. Therefore, the alternative promoter might be more sensitive to changes in CREB activation than the canonical promoter. This fact may explain the differences observed in the expression levels from the canonical and alternative promoters in skeletal muscle at rest and after muscle activity. We cannot ignore the fact that the regulation of transcription from the alternative promoter is sensitive to other CREB-related transcription factors. It has been demonstrated that the transcription factors MyoD and MRF4 can transactivate the alternative promoter through a proximal E-box motif [44].

Through experiments with rodent skeletal muscle, a model of *PGC-1α *gene expression under an acute endurance exercise was proposed [20,48]. A low-intensity exercise does not induce AMPK activation; however, exercise of this type increases the activity of the sympathetic nerve system. As a result, the activation of muscle β2-adrenergic receptors, the accumulation of cAMP, the activation of protein kinase A (PKA), and an increase in the phosphorylation level of CREBSer133 occur (*Fig. 1*). The theoretical AMPK-independent regulation of *PGC-1α *gene expression conforms well with the experimental data. In human skeletal muscle, endurance exercise at a moderate intensity does not increase the phosphorylation level of AMPKThr172 or the expression level of *PGC-1α *from the canonical promoter. Meanwhile, expression of *PGC-1α *derived from the alternative promoter is markedly increased [49,50].

An increase in intense endurance exercise above 50–60% of •Vo_2max_ induces AMPK activation in skeletal muscle [30,31] and increases sympathetic activity. This increase in sympathetic activity activates β-adrenergic receptors in muscle tissue and stimulates expression of the *PGC-1α *gene from the alternative promoter. AMPK activation initiates expression from the canonical promoter. AMPK activation occurs only during high-intensity endurance exercise, which results in substantial muscle metabolic perturbations.

We must emphasize that there is no general consensus concerning the mechanisms of *PGC-1α *gene regulation in skeletal muscle. Several authors have cast doubt on the *PGC-1α *gene regulation model described above. Kim *et al. *investigated the expression of PGC-1α mRNA and protein in rat tissue 6 and 18 h after clenbutelol and noradrenalin injections [55]. These treatments resulted in a marked increase in PGC-1α mRNA levels and protein expression in brown adipose tissue; treatment affected neither gene nor protein expression in skeletal muscle. Clenbuterol treatment resulted in an increased phosphorylation level of CREB (Ser133) in skeletal muscle. However, the activity of luciferase driven by the *PGC-1α *promoter did not change. The authors argued against the above model of *PGC-1α* gene regulation. No increase in the PGC-1α mRNA level was observed using the primer pair designed to be used in the study. The primers complementary to exon 1a (forward) and 2 (reverse) detected only PGC- 1α-a mRNA derived from the canonical promoter; these primers could not detect changes in expression derived from the alternative promoter. The plasmid used to evaluate luciferase activity contained part of the canonical *PGC-1α *promoter, which explains why no increase in clenbutelol-mediated transcriptional activity was observed. However, these aspects of the study do not explain the lack of alteration in the PGC- 1α protein expression observed in skeletal muscle; the antibodies used in the study by Kim *et al. *could detect the proteins encoded by transcripts originating from both promoters.

In another article [49], the effects of AICAR and noradrenalin on cultured human myotubes were evaluated. Treatment with noradrenalin resulted in an increase only in the PGC-1α-b mRNA level; this finding is in agreement with the *PGC-1α *gene regulation model described above. However, AICAR treatment increased both the PGC-1α-a and PGC-1α-b mRNA levels. A combined treatment had an additive effect on expression derived from the alternative promoter. The authors concluded that AMPK is the most important regulator of *PGC-1α *gene expression, since it can regulate expression from both promoters. This finding is in agreement with the result of a recent study in mice [62]. The ability of adrenalin to activate p38 MAPK was demonstrated [56]. This phenomenon could potentially influence *PGC-1α *gene expression from the canonical promoter.

The activation of *PGC-1α *gene expression from different promoters may be regulated by the intensity of the endurance exercise. The aforementioned studies implied that all PGC-1α isoforms are full-length isoforms containing 13 exons. It was demonstrated later that alternative splicing of other PGC-1α mRNA isoforms gives rise to a stop-codon between exons 6 and 7 (see below). Most of the studies cited above used a forward primer that was aligned to one of the first exons (1a, 1b or 1c) and reverse primer that was aligned to the second exon (common to all PGC-1α mRNA isoforms). In most of the studies, PGC-1α protein abundance was evaluated by immunoblotting at a molecular weight greater than 90 kDa (corresponding to the full-length PGC-1α protein). Therefore, the evaluated transcripts encoded both full-length and truncated PGC-1α proteins. These isoforms have different characteristics and functions (see below); many active sites present in fulllength PGC-1α are absent in truncated PGC-1α.

It remains unclear whether all of the PGC-1α mRNA isoforms are translated to proteins *in vivo*; the functions of these hypothetical proteins are also unknown. The N-termini of PGC-1α isoforms differ from each other only by a few amino acids at the beginning of the protein. It is unlikely that such small differences have a substantial influence on the function of these isoforms. The N-terminus often contains sequences related to intracellular transport. Our unpublished data reveal that the N-termini of PGC-1α isoforms do not contain typical nuclear or mitochondrial localization sequences. The absence of known localization sequences does not disprove the hypothesis that isoforms originating from different promoters have a specific intracellular distribution; however, this distribution becomes less probable. The existence of different *PGC-1α *promoters indicates that gene expression is regulated by different signaling pathways activated by different physiological stimuli.

**Truncated PGC-1α isoforms**

In their early study, Baar *et al*. investigated the molecular adaptation of rat skeletal muscle to acute endurance exercise. In a Western blot, increased band intensities were observed for full-length PGC-1α and an additional band at ~34 kDa; it was suggested that this second protein was a smaller form of PGC-1α [57]. Zhang *et al*. have demonstrated that a short insert might appear between exons 6 and 7 as a result of alternative splicing in brown adipose tissue. This insert (exon 7a) contains a stop-codon and encodes an N-truncated (NT) isoform of PGC-1α (*Fig. 2C*). NT-PGC-1α was detected in a Western blot at ~35-38 kDa. An examination of the NCBI nucleotide database uncovered a variant form of PGC-1α mRNA in humans (AB061325) and mice (AB061324) [3]; these sequences encoded proteins 271 and 270 a.a. in length, respectively. In theory, transcription of the NT-PGC-1α isoform can occur from both the proximal (1a) and alternative (1b and 1c) promoters [54]; this may explain the existence of several bands between 35 and 38 kDa [3]. NT-PGC-1α isoforms were found in mice brain tissue and human heart tissue. It is important to note that both the mRNA and protein levels for the full-length and truncated isoforms were comparable [3]. Recent studies have revealed that NTPGC- 1α isoforms are also expressed in human skeletal muscle [52], where they constitute a significant share of total PGC-1α mRNA [50,51].

The NT-isoforms retain the following two essential PGC-1α domains: the N-terminal domain that recruits SRC-1 and CREB-binding proteins and the two LXXLL-like motifs that mediate interactions with nuclear receptors. The NT-isoforms also retain some p38 MAPK, PKA, and AMPK phosphorylation sites. NT-PGC-1α lacks the C-terminal nuclear localization sequence that regulates nuclear targeting, the ligand-independent PPARγ binding region, the SRrich and RRM domains, the FOXO1, MEFC2, and the TRAP220 domains, the C-terminal domain involved in the regulation of protein stability, and multiple sites of post-translation regulation and modification (the GSK- 3β, AMPK, Akt, p38 MAPK, and PKA phosphorylation sites, arginine methylation sites and lysine acetylation sites) [3,58,59]. These marked differences of the NTPGC- 1α isoform compared to full-length isoforms confer it unique properties.

**Intracellular localization and stability**

The intracellular stability and localization of PGC-1α were investigated using cardiomyocyte and COS-7 cultures and mutated PGC-1α proteins lacking various C-terminal fragments [60]. It was demonstrated that the full-length PGC-1α (1-797 a.a.) protein has a short half-life and is mainly localized in the nucleus. A mutant protein containing the amino acids 1–565 localized in the nucleus and cytoplasm. A mutant containing the amino acids 1–292 was found mainly in the cytoplasm. The ablation of C-terminal fragments improved PGC- 1α protein stability. Apparently, this effect is due to a decrease in the ubiquitination level of the protein [60,61]. These findings are related to the properties of the NT-PGC-1α isoforms. The lack of a C-terminal fragment increases the stability of NT-PGC-1α compared to the full-length protein [3,58].

Experiments using a CHO-K1 cell line [3,58] and mice muscle fibers [59] and confocal microscopy have revealed that the NT-isoforms are localized in the cytoplasm (~90%), in contrast to the full-length isoforms, which are localized mainly in the nucleus. A transfection experiment using CHO-K1 demonstrated that the NT-isoforms expressed from both the canonical promoter (NT-PGC-1α-a) and the alternative promoter (NT-PGC-1α-b and NT-PGC-1α-c) are localized in the cytoplasm [54]. These findings confirm that the localization of PGC-1α isoforms depends on the presence of the C-terminal fragment rather than the amino acid sequences encoded by the first exon.

Different proteins regulate the intracellular localization of the NT-isoforms. In murine muscle fibers [59] and in CHO-K1 cells [58], leptomycin B (a specific inhibitor of exportin 1, which is a regulator of nuclear export) increases the NT-PGC-1α level in the nucleus. The authors suggested that the low NT-PGC-1α content in the nucleus depends on the higher rate of exportin 1-mediated nuclear export of NT-PGC-1α compared to the diffusion rate of NT-PGC-1α into the nucleus [58] and possible exportin 1-independent nuclear export [59]. The activation of cAMP-dependent signaling induces an increase in the nuclear NTPGC- 1α content in muscle fibers [59] and in brown adipose tissue [3]. This effect is likely to be regulated by PKA-dependent phosphorylation of NT-PGC-1α at positions 194, 241, and 256; this phosphorylation decreases exportin 1-mediated nuclear export [58]. Conversely, a p38 MAPK-dependent mechanism for the regulation of NT-PGC-1α intracellular localization apparently exists. The inhibition of p38 MAPK tempers the increase in nuclear NT-PGC-1α in brown adipose tissue induced by 8-CPT-cAMP (an analog of cAMP) [3]. However, inhibition of p38 MAPK had only a small negative effect on the increase in nuclear NT-PGC-1α; the inhibition of PKA completely eliminated this increase. These findings suggest that activation of muscle β2-adrenergic receptors regulates intracellular NT-PGC-1α localization. This fact agrees with the results from mice muscle fibers; AICAR-mediated activation of AMPK and the activation of p38 MAPK by electrical stimulation did not increase nuclear NT-PGC-1α [59].

**Regulation of NT-PGC-1α mRNA expression**

The NT-isoforms originate due to the alternative splicing of PGC-1α mRNA, which leads to the formation of a stop-codon between the exons 6 and 7. The expression of the NT-isoforms may be dynamically regulated by different physiological stimuli. Acute endurance exercise initiates comparable increases in the full-length isoform and the NT-isoforms in murine [62] and human skeletal muscle [50,51]. The NT-isoforms can be expressed from the canonical promoter and the alternative promoter [51,62]; the expression magnitude depends on the intensity of the exercise, as observed for full-length isoforms [62].

It can be assumed that expression of both full-length and truncated PGC-1α mRNA isoforms is induced by activation of AMPK and β2-adrenergic receptors. These mechanisms, which regulate mRNA expression, act in the same fashion on both the full-length and truncated isoforms. The expression of both the full-length and truncated PGC-1α mRNA isoforms is upregulated in AICAR-stimulated muscle myotubes [51]. Injection of AICAR and clenbuterol stimulates the expression of both the full-length and truncated isoforms in the skeletal muscles of mice [62]. Conversely, exposure to cold (4°C, 5 h) activates the expression of both NT-PGC-1α and full-length PGC-1α mRNA (~15%) and their corresponding proteins in brown adipose tissue [3]. Under control conditions (22°C), gene expression originates mainly from the canonical promoter (NT-PGC-1α-a and PGC-1α-a mRNA); exposure to cold increases expression from the alternative promoter (NT-PGC-1α-b, NT-PGC-1α-c, PGC-1α-b, and PGC-1α-c mRNA). The latter condition is related to the activation of β2-adrenergic receptors [3,54].

Thom *et al*. have recently demonstrated that hypoxia may induce splicing of PGC-1α mRNA between the exons 6 and 7. Hypoxia (0.5% O_2_, 16 h) increases expression of the NT-isoforms in skeletal muscle myocytes and in myocytes with suppressed HIF-1 and -2 activity. These findings suggest that hypoxia induces splicing of PGC-1α mRNA independent of HIF signaling [63].

The regulation of expression from different promoters and the regulation of splicing between exons 6 and 7 are independent processes; these processes are regulated by different mechanisms. In conclusion, it is unclear whether all of the NT-isoforms can be translated into proteins *in vivo *and whether these hypothetical protein isoforms have different functions.

**Functional roles of NT-isoforms**

Different *in vitro *experimental approaches have clearly demonstrated that NT-PGC-1α is a functionally active protein and can coactivate the following nuclear receptors: PPARα and PPARγ in CHO-K1 cells [3] and PPARα, PPARγ, and ERRα in COS-1 cells [54]. Similar to that for full-length isoforms, overexpression of NT-PGC-1α in brown adipose tissue induces upregulation of UCP1 and CPT-1β mRNA expression and an increased ratio of mitochondrial DNA to nuclear DNA; this ratio serves as a marker of the activation of mitochondrial biogenesis [3].

The function of the NT-isoforms differs significantly from that of the full-length PGC-1α isoforms. The expression of genes targeted by PGC-1α and NT-PGC-1α might differ. Examination of myotubes revealed that overexpression of full-length PGC-1α (PGC-1α-1 in the paper by Ruas *et al.*) alters the expression of 2002 genes, while overexpression of NT-PGC-1α-b (PGC- 1α-4 in paper by Ruas *et al.*) affects the expression of only 519 genes. These isoforms simultaneously influence the expression of only 98 genes [52]. In brown adipose tissue adipocytes expressing PGC-1α or NT-PGC- 1α, the expression of the *Cox7al *and *PPARα *genes increased. However, the increased expression of the* CPT1β, UCP1, ERRα, *and *Cox8b *genes are correlated only with the expression of NT-PGC-1α; *CytC *expression is associated with PGC-1α [58,64].

It has recently been demonstrated that the NT-isoforms predominantly activate angiogenesis, whereas the full-length PGC-1α-a isoforms induce both mitochondrial biogenesis and angiogenesis in skeletal muscle cells [63]. Myotubes derived from *PGC-1α*-/- mice myoblasts were infected with an adenovirus encoding NT-PGC-1α-a or PGC-1α-a. This led to a comparable increase in the mRNA levels of NT-PGC-1α-a and PGC-1α-a; however, the expression of the genes targeted by PGC-1α and their associated proteins differed. In the myotubes expressing NT-PGC-1α-a, the expression of OXPHOS-related genes did not change. The content of complex III and V mitochondrial proteins slightly increased in myotubes expressing NTPGC- 1α-a. In PGC-1α-a infected cells, a pronounced increase in these indices was observed. The same picture was present in the maximal cell respiration rate: this index increased only after PGC-1α-a infection. Conversely, NT-PGC-1α-a induced a more pronounced increase in *VEGFA *gene expression and activation of angiogenesis. Transgenic mice overexpressing the truncated isoform NT-PGC-1α-b (PGC-1α4 in Thom *et al.*) were used to confirm the physiological significance of these findings *in vivo*. In transgenic animals, angiogenesis- related genes (*VEGFA, CD31, ANGPT2*) were expressed at a higher rate than in wild-type animals; capillary density in *m. tibialis anterior *was also greater in transgenic animals. Angiogenesis induced by NT-isoforms might be due to the retained LXXLL motif; this motif can interact with ERRα, which is a regulator of* VEGFA *gene expression [20,63].

Obtaining a knockout of the NT-PGC-1α isoform is a difficult task. Because of this, researchers cannot evaluate the influence of truncated isoforms on the phenotype and function of skeletal muscle and the whole organism. Nevertheless, a few studies [54,65] utilized mice that expressed a mutant PGC-1α-a protein containing the first 254 a.a. (NT-PGC-1α^254^) rather than the full-length PGC-1α protein. The NT-PGC-1α^254^ protein is only a few amino acids shorter than native NT-PGC-1α-a and is a functional equivalent of NTPGC- 1α-a [54,65]. Leon *et al*. [65] showed that the weight of predominantly oxidative *m. soleus *muscle fibers in NT-PGC-1α^254^ mice was slightly lower than that in wild-type animals. However, the weights of predominantly glycolytic *m. tibilas anterior *muscle fibers did not differ between mutant and wild-type mice. A histological examination found no marked changes in the skeletal muscle fibers of NT-PGC-1α^254^ mice. The mitochondrial density, the basal expression of the OXPHOS related genes, the ADP-stimulated maximal respiration rate, the running time to exhaustion during an incremental treadmill test, and the pulmonary •Vo_2max_ were significantly lower in NT-PGC-1α^254^ mice compared with the wild-type control [65]. Conversely, the decrease in the body temperature of adult NT-PGC- 1α^254^ mice was similar to that in the wild-type control after exposure to cold (4°C). In this case, NT-PGC-1α^254^ mice were also able to increase expression of the *UCP1* gene in brown adipose tissue [54,65,66], apparently via the Twist-1-mediated mechanism. It was shown that Twist-1, a negative regulator of full-length PGC-1α, had no effect on the truncated proteins [64].

It is interesting to compare NT-PGC-1α254 mice with mice completely devoid of PGC-1α activity (the PGC- 1α mRNA sequence was changed after exon 2) in either the whole organism [67,68] or in skeletal muscle [69,70]. Whole-body knockout mice did not show any abnormalities in muscle fiber size, fiber composition, and mitochondrial density compared to wild-type animals. The absence of abnormalities could be partially explained by hyperactivity in the knockout mice due to marked abnormalities in the central nervous system [67,68]. In the mice where the *PGC-1α *gene was knocked out in the skeletal muscle, the percentage of oxidative fibers (type I) in the red and white muscles was lower compared to the wild-type control [69,70]. Moreover, knockout mice of both types had noticeably lower basal expression of the OXPHOS related genes in mixed (*m. quadriceps femoris*) and white (*m. gastrocnemius*) muscles compared to the wild-type control [67-70]. In contrast to NTPGC- 1α^254^ mice, adult mice completely lacking PGC-1α activity had a pronounced decrease in body temperature during exposure to cold (4°C) [68]. This effect may be partially explained by the lack of *UCP1 *gene expression in brown adipose tissue, which is mediated by PGC-1α [64]. Taken together, these knockout studies suggest that the functional role of the NT-isoforms differs from that of the full-length isoform.

In this review, the influence of endurance exercise on the regulation of the expression of different PGC-1α isoforms was analyzed. Most studies focused on the effects of acute endurance exercise, because regular aerobic training activates mitochondrial biogenesis and angiogenesis in skeletal muscle. Therefore, the relationship between endurance exercise and PGC-1α seems logical. Recently, Ruas and colleagues demonstrated that the truncated PGC-1α isoform NT-PGC-1α-b (PGC-1α4 in Ruas *et al.*) regulates myogenesis [52]. Myotubes overexpressing NT-PGC-1α-b showed increased mRNA of the growth factor IGF-1 and the myogenic factors Myf-5 and -6; a lower level of myostatin mRNA was observed in myotubes overexpressing NT-PGC-1α-b compared with control cells or cells overexpressing PGC-1α-a. NTPGC- 1α-b-mediated expression of the OXPHOS-related genes was lower than PGC-1α-a-mediated expression. The authors revealed that the NT-isoform, as with full-length PGC-1α, is predominantly localized in the nucleus. This finding does not agree with the previous studies of intracellular localization of NT-PGC-1α [58,59]. Overexpression of NT-PGC-1α-b by both adenovirus injection and plasmid electroporation significantly increases the expression of the truncated protein, the area of fiber cross sections, and the weight of mouse muscles compared to those in wild-type animals. Electroporation of the plasmid encoding the truncated isoform (NT-PGC-1α-a) driven by the canonical promoter increased the NT-PGC-1α-a mRNA level; however, increased translation of the truncated protein was not observed in a Western blot. The authors concluded that the N-terminal amino acid sequence of NT-PGC-1α-b allows for the accumulation of this protein in the cell; this sequence is missing in NT-PGC-1α-a [52]. This finding is not in agreement with the experiment in which the level of the truncated protein increased in myotubes infected with adenovirus-encoded NT-PGC-1α-a [63]. The physiological significance of NT-PGC-1α-b overexpression was investigated using transgenic mice. A small increase in mRNA expression of VEGFA, EERα, myoglobin mRNA was observed in transgenic mice; a decrease in myostatin mRNA expression and no changes in the mRNA expression of IGF-1 and other myogenic regulators was also observed compared to wild-type animals [52,63]. The area of muscle fiber cross sections, the muscle weight and force, and the running time to exhaustion during treadmill test were slightly higher in transgenic animals compared to control mice [52]. In the cited study, the effect of acute exercise on the expression of NT-PGC-1α-b mRNA was not investigated. However, the basal expression level of this transcript in human skeletal muscle was shown to increase after 8 weeks of strength training and to be unchanged after 8 weeks of endurance training. A primer pair aligning to exons 5 (forward) and 7a (reverse) was used to detect NT-PGC-1α-b mRNA in this study. This primer pair can detect both NT-PGC-1α-b and NT-PGC-1α-a transcripts. A recent study has demonstrated that acute strength training and endurance exercise induce the expression of both isoforms in human skeletal muscle [51]. Therefore, it is possible that the strength training that occurred in the Ruas *et al*. study [52] may have induced expression of both NT-PGC-1α-b and NT-PGC-1α-a mRNA.

The influence of PGC-1α isoforms expressed from the canonical promoter on skeletal muscle hypertrophy was investigated using synergist ablation [71]. An increase in the absolute phosphorylation level of mTORC1 targets, increased IGF-1 mRNA abundance, and a decrease in the myostatin mRNA level in hypertrophied muscle were observed as compared to the control muscle. Moreover, PGC-1α mRNA expression from the alternative promoter (PGC-1α-b and NT-PGC-1α-b, detected using a primer pair aligning to exons 1b and 2) and the canonical promoter (PGC-1α-a and NT-PGC-1α-a, detected using a primer pair aligning to exons 1a and 2) decreased; the expression of the OXPHOS related genes and the content and activity of key mitochondrial proteins also decreased. Following synergist ablation,* PGC-1α *knockout mice showed a comparable increase in muscle weight, an absolute phosphorylation level of mTORC1 targets, and an IGF-1 mRNA level, as well as a decrease in myostatin mRNA abundance compared to wild-type animals after synergist ablation. The authors draw a conclusion that PGC-1α is not involved in the chronic overload-induced remodeling of skeletal muscle. This conclusion indirectly supports the hypothesis that the expression of NT-PGC-1α-b mRNA is regulated by the same stimuli as those that regulate the expression of PGC-1α-b mRNA; these stimuli are exercise intensity and clenbuterol-mediated activation of β2-adrenergic receptors [62]. In conclusion, these studies demonstrated that the influence of the PGC-1α isoforms on the mechanisms of protein synthesis are not fully clear and require further investigation.

## CONCLUSIONS

The coactivator PGC-1α is a key regulator of mitochondrial biogenesis, fat and carbohydrate metabolism. Both *in vitro *and *in vivo *studies have demonstrated that several isoforms of PGC-1α mRNA may be expressed in rodent and human skeletal muscle. The expression patterns may markedly differ at rest and after muscle activity. Different signaling pathways are activated by different physiological stimuli that regulate the expression of the *PGC-1α *gene from different promoters. Apparently, the expression from the canonical (proximal) promoter is regulated mainly by the activation of AMPK, while expression from an alternative promoter is regulated via the β2-adrenergic receptor. Most probably, the functional properties of isoforms derived from different promotors do not differ. Therefore, the availability of two signaling pathways regulating the *PGC-1α *gene expression provides ample opportunities for a pharmacological influence on the expression of this gene. Those opportunities might be important in treating and preventing various diseases, such as metabolic syndrome and diabetes mellitus.

All transcripts, from both the canonical and alternative promoters, are subject to alternative splicing. As a result, truncated isoforms that possess different properties are translated. The truncated isoforms are more stable and predominantly activate angiogenesis, whereas full-length isoforms regulate manly mitochondrial biogenesis. It has recently been shown [52] that in contrast to full-length isoforms, truncated isoforms may regulate myogenesis, but this assumption needs further confirmation. The existence of several isoforms with a broad-spectrum of functions allows the organism to adapt to different physiological stimuli.

The mechanisms of *PGC-1α *gene expression in human skeletal muscle remain not fully clear. Elucidation of the regulatory mechanisms of *PGC-1α *gene expression and their functional role may provide an opportunity to control the expression of different isoforms through exercise and/or pharmacological interventions. This opportunity is important for patients with the metabolic syndrome and diabetes mellitus and perhaps for endurance athletes.

## Abbreviations

AICAR: 5-aminoimidazole-4-carboxamide-1-β-D-ribofuranosyl 5′-monophosphate
AMPK: AMP-activated protein kinase
ATF: activating transcription factor
CaMK: Ca2+/calmodulin-dependent protein kinase
CREB: cAMP response element-binding protein
ERR: estrogen-related receptor
HDAC: class IIa histone deacetylase
HIF: hypoxia inducible factor
IGF-1: insulin-like growth factor 1
MEF: myocyte enhancer factor
OXPHOS: oxidative phosphorylation
p38 MAPK: p38 mitogen-activated protein kinases
PGC: peroxisome proliferator-activated receptor gamma, coactivator
PKA: protein kinase A
PPAR: peroxisome proliferator-activated receptor
UCP: uncoupling protein
VEGFA: vascular endothelial growth factor A
•Vo2max: maximal oxygen consumption rate
